# Adenosine 5′-Triphosphate Metabolism in Red Blood Cells as a Potential Biomarker for Post-Exercise Hypotension and a Drug Target for Cardiovascular Protection †

**DOI:** 10.3390/metabo8020030

**Published:** 2018-05-02

**Authors:** Pollen K. Yeung, Shyam Sundar Kolathuru, Sheyda Mohammadizadeh, Fatemeh Akhoundi, Brett Linderfield

**Affiliations:** Pharmacokinetics and Metabolism Laboratory, College of Pharmacy and Department of Medicine, Dalhousie University, Halifax, NS B3H 4R2, Canada; shyam.sundar.kolathuru@dal.ca (S.S.K.); sheyda.moh@dal.ca (S.M.); ft714365@dal.ca (F.A.); Brett.Lindenfield@dal.ca (B.L.)

**Keywords:** ATP, adenosine, metabolism, exercise, cardiovascular protection, drug target

## Abstract

The importance of adenosine and ATP in regulating many biological functions has long been recognized, especially for their effects on the cardiovascular system, which may be used for management of hypertension and cardiometabolic diseases. In response to ischemia and cardiovascular injury, ATP is broken down to release adenosine. The effect of adenosine is very short lived because it is rapidly taken up by erythrocytes (RBCs), myocardial and endothelial cells, and also rapidly catabolized to oxypurine metabolites. Intracellular adenosine is phosphorylated back to adenine nucleotides via a salvage pathway. Extracellular and intracellular ATP is broken down rapidly to ADP and AMP, and finally to adenosine by 5′-nucleotidase. These metabolic events are known to occur in the myocardium, endothelium as well as in RBCs. Exercise has been shown to increase metabolism of ATP in RBCs, which may be an important mechanism for post-exercise hypotension and cardiovascular protection. The post-exercise effect was greater in hypertensive than in normotensive rats. The review summarizes current evidence in support of ATP metabolism in the RBC as a potential surrogate biomarker for cardiovascular protection and toxicities. It also discusses the opportunities, challenges, and obstacles of exploiting ATP metabolism in RBCs as a target for drug development and precision medicine.

## 1. Introduction

**Figure 1:**
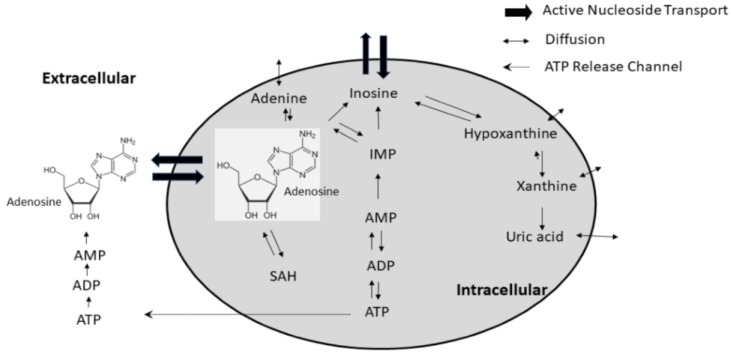
Complex ATP and adenosine metabolism. Abbreviation: SAH = *S*-adenosyl homocysteine.

The importance of adenosine (ADO) and adenosine 5′-triphosphate (ATP) in regulating many biological functions has long been recognized, especially for their effects on the cardiovascular system [1,2,3,4,5,6,7,8,9,10]. It is known that ADO and ATP are key factors in the regulation of coronary blood flow [5,11,12,13], inhibiting platelet aggregation [14], protection of myocardium [10,15,16,17], neuromodulation [18,19,20,21,22,23,24,25], attenuating tissue necrosis [7,26], ischemic preconditioning [27,28,29,30,31,32], immunomodulation [33,34], energy metabolism [9,35,36,37,38], cancer biology signaling [39], and perhaps other functions as well (e.g., pain mediation), which maintain homeostasis of the cardiovascular system. Under normal physiological conditions, the main source of intracellular ADO is from hydrolysis of *S*-adenosylhomocysteine, and from catabolism of ATP to adenosine 5′-diphosphate (ADP) and then to adenosine 5′-monophosphate (AMP), which is further catabolized by ecto and endo 5′ nucleotidase to produce ADO [38,40,41]. Extracellular concentrations of ADO, such as those found in plasma under normal physiological conditions, are kept very low (uM range or below) because of rapid uptake by active nucleoside transporters throughout the vasculature, and also by catabolism to other oxypurine metabolites, such as hypoxanthine and uric acid [42,43,44]. However, during ischemia/hypoxia or in extremely heavy workloads, such as intense exercise, there is an increased demand of energy, which triggers a rapid breakdown of ATP and release of ADO locally and into systemic circulation [43,45]. After restoring the hypoxia to normal physiologic conditions, the released ADO is taken up rapidly by endothelial cells and RBC via the nucleoside transporters, and subsequently converted back to ATP by ADO and adenylate kinases [46,47]. Such a salvage pathway is capable of recycling ATP from ADO readily, even in cells without mitochondria, such as the RBC [48,49]. Inhibitors of nucleoside transporters, such as dipyridamole, can prolong the action of adenosine, and may potentially interrupt recycling of ADO to ATP via the salvage pathway. A schematic diagram illustrating the transport and metabolism of ATP and ADO highlighting the salvage pathway is summarized in Figure 1.

Biomarkers are increasingly used in drug discovery and development, and also in disease management [50]. Based on the definition proposed by the FDA Biomarker Definition Working Group [51], a “Biomarker is a characteristic that is objectively measured and evaluated as an indicator of normal biological processes, pathogenic processes, or pharmacologic responses to a therapeutic intervention”. It has been suggested ADO and ATP catabolism may be used as biomarkers to quantify myocardial and endothelial ischemia [1,52,53], cardiovascular toxicities [43], and as a potential target for anti-ischemia drugs [54,55,56,57,58]. On the other hand, exercise has been shown to improve cardiovascular hemodynamic and increase RBC concentrations of ATP in both humans and animal models [59,60]. The increase of circulatory concentrations of ADO and ATP are believed to be key factors for exercise preconditioning and post-exercise hypotension, and could be a mechanism responsible for cardiovascular protection [27,43,61,62]. The review summarizes currently available evidence in support of ATP metabolism in the RBC as a potential systemic biomarker for cardiovascular protection and toxicities. It also discusses the opportunities, challenges, and obstacles of exploiting ATP metabolism in RBCs as a target for drug development and precision medicine.

## 2. Biomarker for Cardiovascular Toxicities

**Figure 2:**
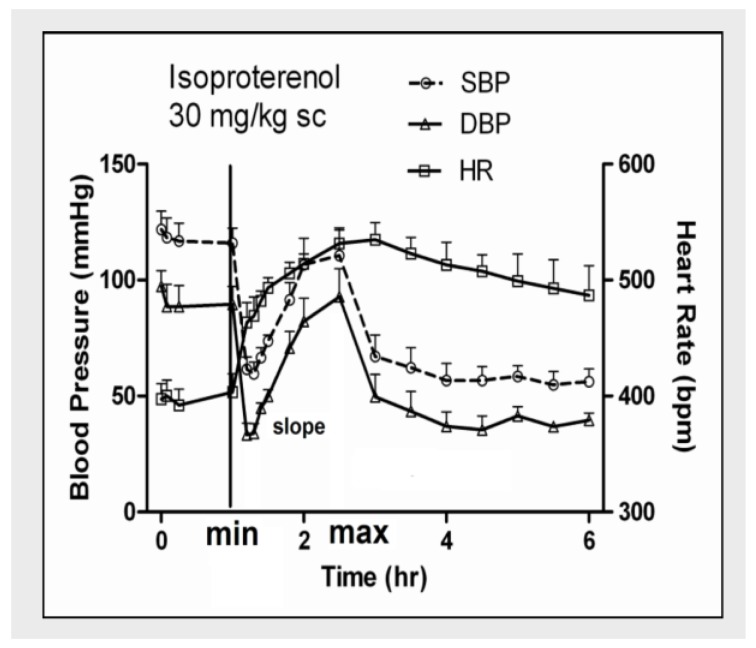
Hemodynamic effect before and after isoproterenol injection (30 mg/kg) in rats. Each point represents mean ± SEM.

**Figure 3:**
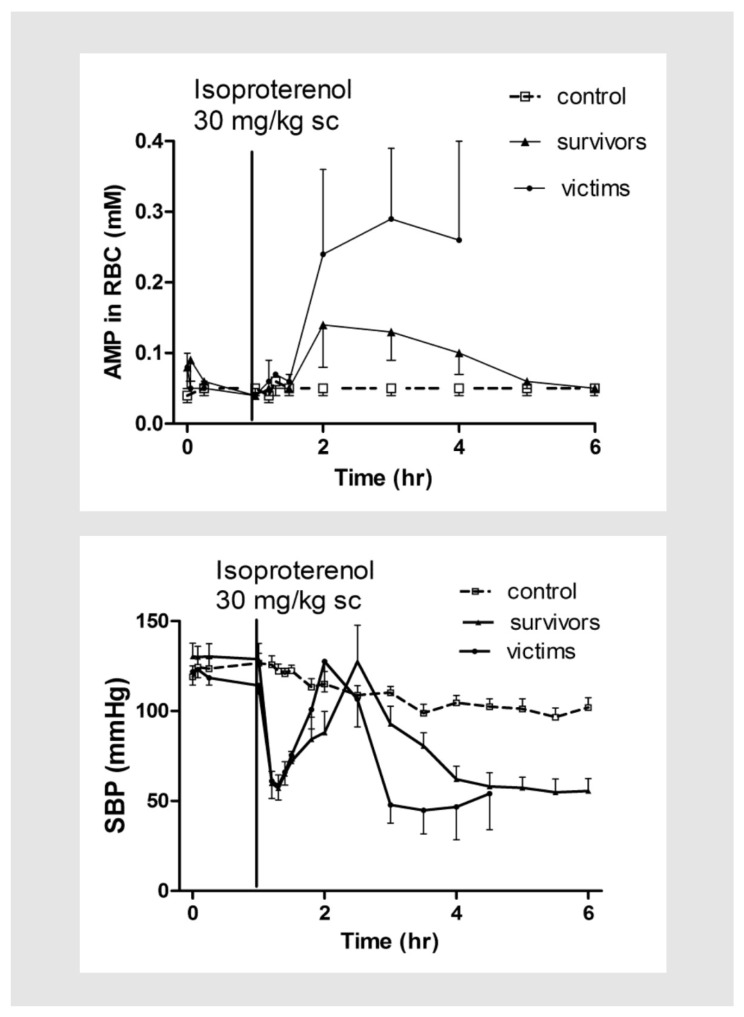
Effect of isoproterenol injection (30 mg/kg) on concentrations of AMP in RBCs and SBP. Each point represents mean ± SEM. Please note survivors were rats which survived from the experiment; victims died in less than 5–6 h after isoproterenol; and control did not receive isoproterenol.

In an experimental rat model of acute myocardial infarction, a single dose of 30 mg/kg of isoproterenol, together with repeated blood sample collection, was shown to induce approximately 50% mortality within 5–6 h after the subcutaneous injection (*p* < 0.05) [63]. It decreased blood pressure (both SBP and DBP) immediately after the injection (<15 min) by greater than 50 mmHg, and increased heart rate (HR) by over 150 bpm towards the end of the experiment (*p* < 0.05). Both SBP and DBP rebounded close to pretreatment (baseline) levels 1–2 h after the injection (*p* < 0.05), and then fell again for the remainder of the experiment. There was no rebound from the HR response (Figure 2). In addition, isoproterenol also increased RBC concentrations of ADP and AMP immediately after injection (*p* < 0.05), with a corresponding decrease of ATP concentration, although the decrease was not statistically significant. The dead rats (victims) had greater increase of AMP and ADP concentrations than the surviving ones (survivors) after isoproterenol, and the rebound in blood pressure was also more acute in the dying rats, suggesting that the rebound in blood pressure and breakdown of ATP in the RBC are serious signs of cardiovascular toxicities, and could be interrelated (Figure 3) [63]. In addition to breakdown of ATP in the RBC, cardiovascular toxicities induced by isoproterenol are also associated with increased plasma concentrations of ADO and uric acid, which is attributed mainly to increased breakdown of ATP to ADO, and also, increased catabolism of ADO to uric acid. However, it was shown that breakdown of plasma ADO was less predictive for cardiovascular mortality than breakdown of ATP in the RBC [43]. It is possible breakdown of ATP in the RBC is a measure of systemic cardiovascular toxicities, while catabolism of ADO to oxypurine metabolites, such as hypoxanthine and uric, as found in the plasma samples, was a measure of catabolism of ATP in both RBCs, as well as from other tissues in the body. Similarly, acute toxicities induced by repeated injection of doxorubicin (10 mg/kg) twice daily for 4 doses also increased AMP concentrations in the RBC, and plasma concentrations of uric acid, although the increase was significantly less than after isoproterenol, suggesting a lesser breakdown of RBC ATP to AMP, and ADO to uric acid, after doxorubicin [64]. The fact that only one of the eight rats died after doxorubicin vs 50% mortality from isoproterenol further supports our working hypothesis that ATP breakdown in the RBC is a serious cardiovascular event, and may be exploited as a surrogate biomarker for cardiovascular mortality.

## 3. Biomarker for Post-Exercise Hypotension and Cardiovascular Protection

Exercise is increasingly used as an adjunct therapy to improve cardiovascular health in complementary and preventive medicine [65]. Post-exercise hypotension (PEH) is a phenomenon describing a prolonged decrease of the resting blood pressure in the minutes and hours following acute exercise [66]. The mechanism of PEH and its relationship with cardiovascular protection is not fully understood. Exercise is considered a form of ischemic preconditioning, and individuals who exercise regularly would have a smaller infarct size and overall improved left ventricular systolic function when encountering a clinical ischemic event, such as an acute myocardial infarction MI [67]. The beneficial effects of exercise preconditioning or post-exercise hypotension on endothelial function and the myocardium may be mediated via increased blood flow, enhanced nitric oxide production and bioavailability, changes in neurohormone release, improvements in oxidant/antioxidant balance, and optimization of energy and ATP metabolism [68,69,70,71]. It is likely that many, or all, of these factors contribute to the cardiovascular health benefits from exercise.

**Figure 4:**
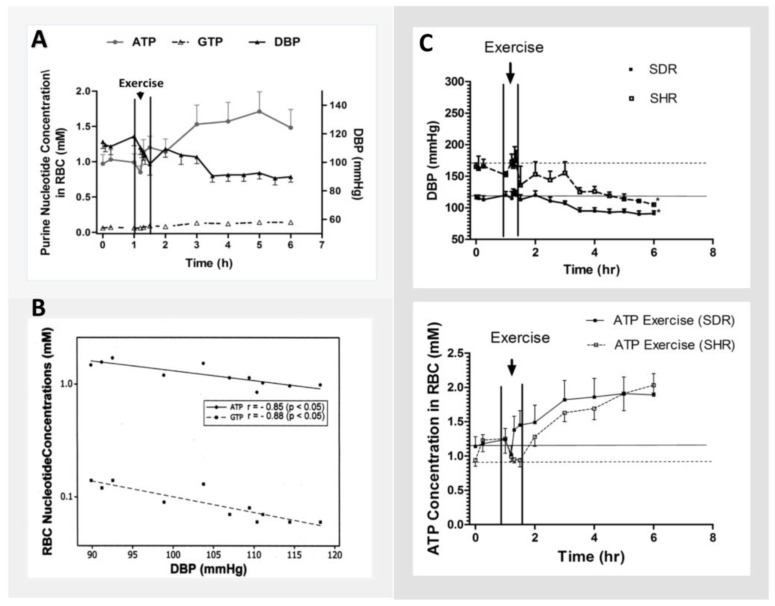
Effect of exercise on hemodynamics and RBC concentrations of purine nucleotides in normotensive Sprague Dawley rats (SDR), and spontaneously hypertensive rats (SHR). Each point represents mean ± SEM for (**A**,**C**). For (**B**), each point represents mean value of 9 SDR. Abbreviations: ATP = Adenosine 5′-triphosphate; GTP = guanosine 5′-triphosphate.

Dudzinska and colleagues [59] have shown in healthy subjects that exercise increased production of ATP in the RBC. The effect was specific mainly to adenine nucleotide metabolism and did not affect metabolism of guanine and pyridine nucleotides in the RBC. In an experimental rat model, we have shown a moderate level of exercise for 15 min at a speed of 10 m/min with a 5% gradient on a treadmill increased RBC concentrations of ATP and guanosine-5′-triphosphate (GTP) for 5 h post exercise (*p* < 0.05 for both), which were highly correlated with PEH (Figure 4) [71]. The exercise effect on increasing purine nucleotide metabolism appeared to be greater for RBC concentrations of ATP, than GTP (Figure 4), which was similar to the effect found in humans [59]. It is interesting to note while the hemodynamic response to exercise was similar between the normotensive Sprague Dawley (SD) rats and the spontaneously hypertensive rats (SHR), the post-exercise effect was significantly greater in the SHR (Figure 4) [60]. Although the reason for the difference is not clear, we hypothesize it could be attributed to a much greater basal energy demand for ATP, resulting in a lower ATP reserve in the SHR. In response to exercise, which further increases energy demand, the concentrations of ATP in the RBC actually fell during exercise in the SHR, but not in the SDR (Figure 4). However, the ATP concentration in the RBC increased sharply following exercise (Figure 4) which contributed, at least in part, to the more profound post-exercise effect [60]. It is possible the SHR could be more vulnerable to ischemia attack because of the lower energy reserve, which will be an exciting hypothesis for further investigation.

**Figure 5:**
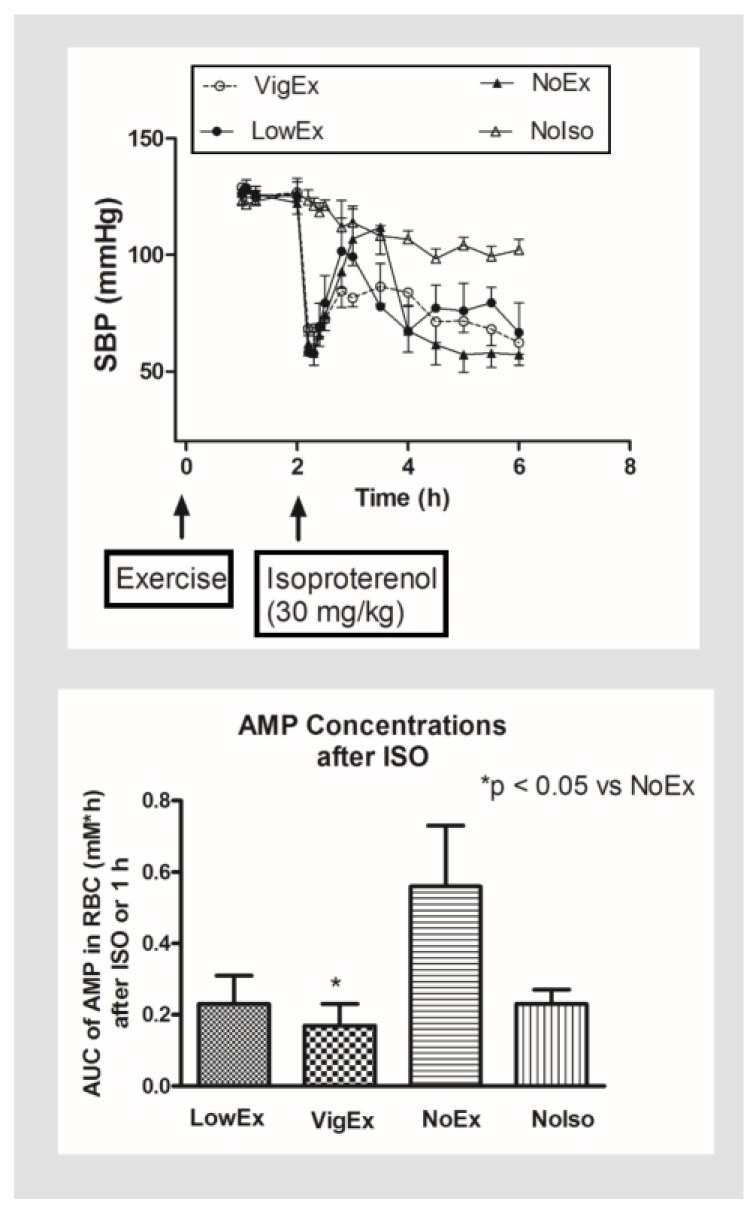
Effect of acute exercise preconditioning on systolic blood pressure (SBP) (**top**) and RBC concentrations of AMP (**bottom**). Each point represents mean ± SEM. (Abbreviations: VigEx = 14 m/min and 22% gradient; LowEx = 10 m/min and 10% grade; NoEx = No exercise; and NoIso = No isoproterenol.

The potential of cardiovascular protection from exercise has been reported by many investigators. It has been shown a single bout of swimming exercise sustained left ventricular function after isoproterenol-induced injury in mice, which lasted over 4 weeks after the exercise [72]. Similarly, treadmill exercise has also been shown to have prolonged cardiovascular protective effects against injury induced by isoproterenol in rats [73]. In patients with stable systolic heart failure, exercise training can relieve symptoms, improve functional capacity and quality of life, as well as reduce the length of hospitalizations, and all-cause mortality [74,75]. Exercise has also been used as an adjunctive therapy to reduce cardiovascular complications in chronic disease, and also to reduce cardiotoxicity associated with anthracycline anticancer agents [76,77]. Conversely, lack of exercise or physical inactivity is a significant cardiovascular risk factor [78,79], and frequent or long-term vigorous exercise may also postpose damaging cardiovascular adverse effects [80]. We have shown in an experimental rat model that preconditioning rats with acute exercise on a treadmill for 15 min at either moderate (LowEx 10 m/min and 10% gradient) or more intense levels (VigEx 14 m/min and 22% gradient) 2 h before isoproterenol reduced mortality, attenuated the rebound in blood pressure, and reduced the production of AMP secondary to breakdown of ATP in the RBC. The protective effect was more apparent in the rats preconditioned with the more vigorous exercise (VigEx) (Figure 5) [62]. Thus, we hypothesize that the cardiovascular protective effect of exercise and the post-exercise effect is mediated by increased metabolism and production of ATP in RBCs and other cell types, which would preserve, either directly or indirectly, intracellular ATP concentration when encountering an insult induced by oxidative stress and/or cardiovascular injury. It is possible that RBC concentrations of ATP may be an indicator of the concentrations in the myocardium and other tissues. It has been hypothesized that RBC serves as an oxygen sensor in the cardiovascular system [81,82,83] which is capable of releasing increased amounts of ATP as oxygen content falls, and its hemoglobin becomes desaturated [84]. Thus, when RBC travels through microcirculation, it could release vasodilatory compounds, such as ATP and adenosine, that enhance blood flow in hypoxic tissues [82]. The released ATP and adenosine would help to increase blood supply to the tissue and preserve an optimum balance between oxygen supply and demand, thereby maintaining cardiovascular homeostasis and preserving intracellular ATP in the tissues affected. Such a mechanism would eliminate the requirement for a diverse network of sensing sites throughout the vasculature, and should provide a more efficient means of appropriately matching oxygen supply with demand, and allow an immediate switch to an alternative energy sources under hypoxia conditions [85]. Thus, it is possible that ATP metabolism in the RBC may be used as a surrogate biomarker for energy content in the myocardium, and perhaps also as a measure of the “inner energy” in the body.

While the currently available data for ATP metabolism in the RBC as a potential surrogate biomarker for cardiovascular protection and toxicity (Section 2) are mostly reported from animal experiments, for ethical and methodological reasons, the beneficial effect of exercise and exercise preconditioning on lowering cardiovascular risk factors, as demonstrated in many clinical trials, is undeniable [86,87,88,89]. It is probable a key mechanism for post-exercise hypotension is via an increase in ATP metabolism in the RBC, which if proven correct, will provide a surrogate biomarker for developing novel strategy for cardiovascular prevention and protection.

## 4. Biomarker and Target for Development of Drug Therapy

**Figure 6:**
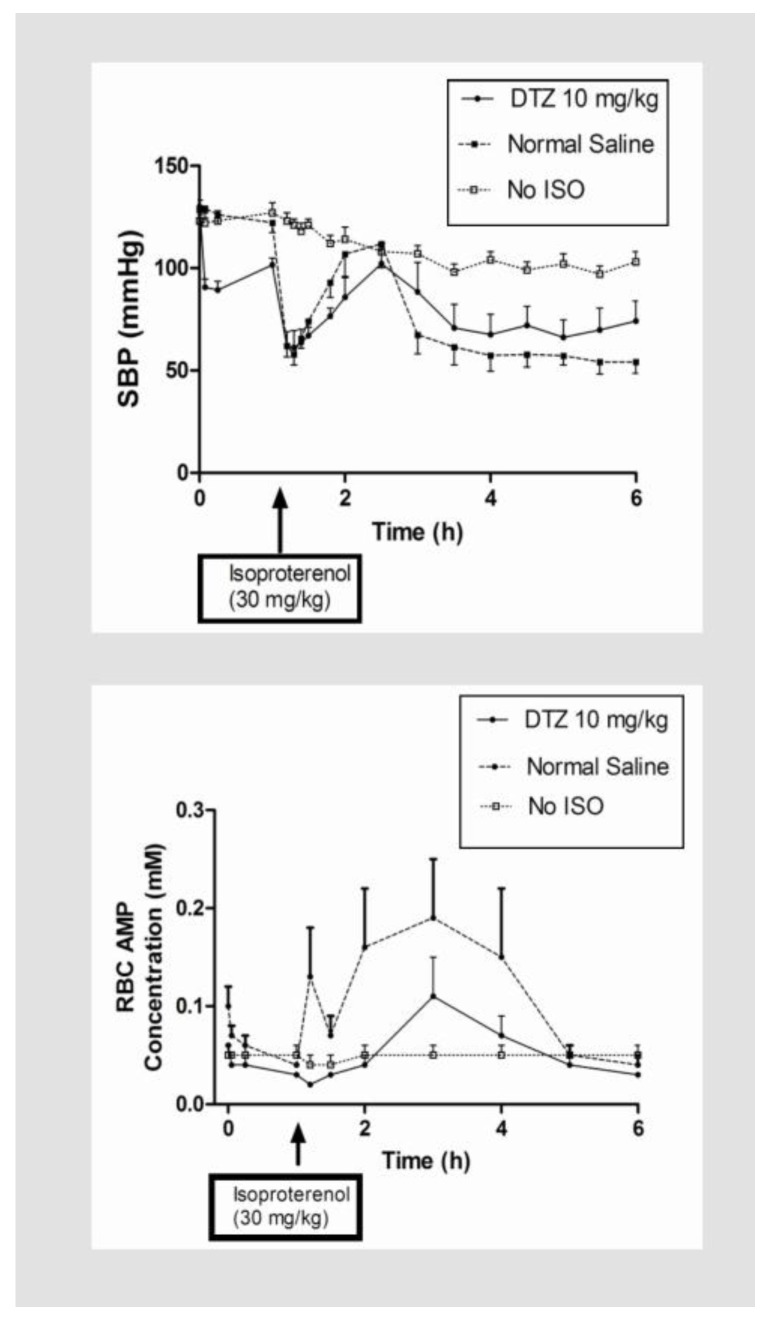
Effect of diltiazem (DTZ) on systolic blood pressure (SBP) (**top**) and RBC concentrations of AMP (**bottom**). Each point represents mean ± SEM. (Abbreviations: No ISO = No isoproterenol).

It has been suggested that ATP catabolism in the RBC may be used as biomarker to quantify myocardial and endothelial ischemia [1,52], and as a potential target for anti-ischemia drugs [54,55,56,57,58]. We hypothesize agents which preserve ATP concentrations in the RBC would have protective effects against ischemia and/or cardiovascular injury [90]. Using the calcium channel blocker diltiazem (DTZ) as a probe, a notable protective effect against cardiovascular injury induced by isoproterenol was demonstrated after 10 mg/kg dose, given by subcutaneous injection twice daily for 5 doses. Specifically, it reduced mortality from 50% to <20%, attenuated the rebound in blood pressure (both SBP and DBP), and breakdown of ATP in the RBC (Figure 6). There was no effect on the increased heart rate, and the protective effect was dose dependent, as it was not observed after a 5 mg/kg dose [58]. Thus, the results suggest the protective effect of DTZ could be measured by reduction of the blood pressure rebound, and breakdown of ATP in the RBC induced by isoproterenol. It should be noted that apart from lowering blood pressure, DTZ also has an appreciable effect on inhibiting exogenous adenosine uptake by RBC [91], which could prolong the anti-ischemia effect of adenosine after release. A similar protective effect was also observed for the classical adenosine reuptake inhibitor dipyridamole [92]. However, it is not clear how blocking adenosine reuptake could preserve ATP concentrations and reduce its breakdown in the RBC in vivo, which could be a key factor associated with the protective effect of these cardiovascular agents. Preliminary studies in our laboratory have shown natural antioxidants, such as coenzyme Q10 and tetracycline antibiotics known to preserve ATP concentration in macrophages in an experimental sepsis model [93], also increase ATP concentrations in RBCs in rats [94,95]. Thus, it is possible ATP metabolism in the RBC may be used as a drug target to identify potential therapeutic agents for cardiovascular protection, cardiometabolic diseases, antibiotics suitable for sepsis management, and anticancer agents with less cardiovascular adverse effects, and as a surrogate biomarker for energy metabolism in the body for the management of chronic diseases.

## 5. Challenges, Opportunities, and Future Direction

There is increasing evidence that ATP metabolism in the RBC has potential as a surrogate biomarker for cardiovascular protection, and could be exploited as a therapeutic target for drug development and in precision medicine [50]. However, study of ATP metabolism in the RBC has been hampered by the inherent instability of ATP and adenosine in whole blood, which requires collection of blood samples in a stopping solution to prevent in vitro breakdown and production of ATP and adenosine [55,96,97,98,99]. A typical stopping solution should consist of a suitable amount of an adenosine uptake inhibitor, such as dilazep or dipyridamole, an inhibitor of adenosine demethylase, such as erythro-9-(2-hydroxyl-3-nonyl) adenine (EHNA), indomethacin to block release of ATP, and EDTA to prevent hemolysis. However, each laboratory should carry out separate experiments to validate the blood collection method prior to the experiment. We have found even with the stopping solution, blood samples still have to be processed immediately after collection to separate plasma from the RBC before storage. In addition, blood samples should be collected via a polyethylene catheter to avoid damage to the blood cell components. These required procedures have restricted clinical studies mainly to a research setting.

Despite the methodological challenge, we believe ATP metabolism in the RBC is still adoptable to chronic disease management as a measure of inner energy, reserves, and cardiovascular homeostasis for prevention, diagnosis, and treatment of cardiometabolic diseases, cancer, aging, stroke, and other neurodegenerative diseases. It could also be exploited as a drug target for the development of cardiovascular protective agents (e.g., anti-hypertensive and anti-platelet agents), anticancer drugs with less cardiotoxicity, antibiotics for sepsis, and anti-inflammatory agents. Finally, it could also be used to investigate the effectiveness of antioxidants as a preventive strategy in alternative and complementary medicines.

## 6. Concluding Remarks

In conclusion, we believe ATP metabolism in RBCs is a potential biomarker for post-exercise hypotension and cardiovascular protection. Breakdown of ATP in the RBC is a sign of serious cardiovascular toxicity and/or mortality. Preserving ATP in the RBC is a potential therapeutic target for cardiovascular protection, which may be used as a surrogate to measure inner energy, reserves, and cardiovascular homeostasis.
